# Unexpected high vulnerability of functions in wilderness areas: evidence from coral reef fishes

**DOI:** 10.1098/rspb.2016.0128

**Published:** 2016-12-14

**Authors:** Stéphanie D'agata, Laurent Vigliola, Nicholas A. J. Graham, Laurent Wantiez, Valeriano Parravicini, Sébastien Villéger, Gerard Mou-Tham, Philippe Frolla, Alan M. Friedlander, Michel Kulbicki, David Mouillot

**Affiliations:** 1 MARBEC, UMR IRD-CNRS-UM-IFREMER 9190, Université Montpellier, 34095 Montpellier Cedex, France; 2 ENTROPIE, UMR IRD-UR-CNRS 9220, Laboratoire d'Excellence LABEX CORAIL, Institut de Recherche pour le Développement, BP A5, 98848 Nouméa Cedex, New Caledonia; 3 Wildlife Conservation Society, Marine Programs, 2300 Southern Boulevard, Bronx, NY 10460, USA; 4 Australian Research Council Centre of Excellence for Coral Reef Studies, James Cook University, Townsville, Queensland 4811, Australia; 5 Lancaster Environment Centre, Lancaster University, Lancaster LA1 4YQ, UK; 6 Université de Nouvelle Calédonie—Laboratoire « LIVE » EA4243, BP R4–98851, Nouméa, New Caledonia; 7 Ecole Pratique des Hautes Etudes, USR 3278 EPHE-CNRS-UPVD CRIOBE, University of Perpignan, 66860 Perpignan Cedex, France; 8 Entreprise Générale de Logistique Environnementale (EGLE SARL), Tribu de Fatanaoué, 98833 Voh-Temala, New Caledonia; 9 Fisheries Ecology Research Lab, University of Hawaii, 2538 McCarthy Mall, Honolulu, HI 96822, USA; 10 Pristine Seas, National Geographic Society, 1145 17th Street NW, Washington, DC 20036, USA; 11 ENTROPIE, UMR IRD-UR-CNRS 9220, Laboratoire d'Excellence LABEX CORAIL, Institut de Recherche pour le Développement, University of Perpignan, 66860 Perpignan Cedex 9, France

**Keywords:** coral reef fish, wilderness areas, redundancy, baseline functional vulnerability

## Abstract

High species richness is thought to support the delivery of multiple ecosystem functions and services under changing environments. Yet, some species might perform unique functional roles while others are redundant. Thus, the benefits of high species richness in maintaining ecosystem functioning are uncertain if functions have little redundancy, potentially leading to high vulnerability of functions. We studied the natural propensity of assemblages to be functionally buffered against loss prior to fishing activities, using functional trait combinations, in coral reef fish assemblages across unfished wilderness areas of the Indo-Pacific: Chagos Archipelago, New Caledonia and French Polynesia. Fish functional diversity in these wilderness areas is highly vulnerable to fishing, explained by species- and abundance-based redundancy packed into a small combination of traits, leaving most other trait combinations (60%) sensitive to fishing, with no redundancy. Functional vulnerability peaks for mobile and sedentary top predators, and large species in general. Functional vulnerability decreases for certain functional entities in New Caledonia, where overall functional redundancy was higher. Uncovering these baseline patterns of functional vulnerability can offer early warning signals of the damaging effects from fishing, and may serve as baselines to guide precautionary and even proactive conservation actions.

## 1. Introduction

Human activities have already induced the collapse of many ecosystems around the world [1–3] and, in combination with climate change, have triggered major reductions in biodiversity globally [3–7]. Beyond the loss of species, there is a growing awareness that the loss of ecological functions may be the most critical consequence of human disturbances on ecosystems [8–12]. This diversity of ecological functions sustains ecosystem services on which humanity depends; such as biomass production [10]. Sustaining ecosystem functions requires both high functional diversity, i.e. a large breadth of ecological functions supported by species [13–17], and high functional redundancy, i.e. a large number of species supporting identical functions in the system [18].

In theory, species richness is thought to maintain a high level of both functional diversity and redundancy, thus ensuring the long-term functioning of ecosystems in a fluctuating environment [19]. Indeed, high species richness should increase the probability of having both species supporting different functions (functional diversity) and many species supporting the same ecological functions (functional redundancy) [17]. Many experiments confirm this theory, for example demonstrating the vulnerability of ecosystem functioning to species loss [11,20]. In natural systems, however, the benefits of high species richness to maintaining ecosystem functioning have recently been challenged by three patterns. First, at the scale of ocean basins (and using species checklists), some functions exhibit over-redundancy, i.e. are supported by a disproportionately high number of species, while others are realized by few or one species only, even in the richest regions [21]. Second, species that support specific or unique traits in ecosystems (no redundancy) tend to be rare owing to their low abundance in ecosystems [22]. Third, the distribution of species richness and abundance among trophic groups is more critical than simply the number of species to maintain ecosystem functioning and services [23]. These patterns demonstrate the importance of preserving both species and abundance within a wide range of functional groups.

Taken together, these results suggest that high levels of species richness and abundance may not insure ecosystems against functional diversity loss as we once hoped, owing to the high vulnerability of some functions that lack redundancy. Yet, this hypothesis has, to our knowledge, never been tested with empirical data in tropical ecosystems with marginal or no exposure to threats, i.e. where species density and abundances should be close to natural baselines. Assessing the vulnerability of ecological functions to threats in such scenarios would reveal the baseline of functional vulnerability, and the extent to which these ecosystems are buffered against even limited local species declines or extinctions.

Protected areas (PAs) are often used to assess ecological baselines against which biodiversity levels in exploited areas are compared [24]. However, recent studies have shown that even PAs cannot be considered as true ecological baselines because anthropogenic disturbances typically started long before these areas were established [2,25–27]. In addition, most of these areas are either too small or embedded in areas influenced by human activities and therefore cannot support the full range of ecological functions [24,28]. As an alternative, wilderness areas, i.e. large areas geographically isolated from humans by natural geographical barriers or with very limited human presence, may provide ecological baselines close to a ‘natural’ status [2,26,28]. Indeed, wilderness areas are traditionally viewed as areas featuring exceptional concentrations of biodiversity and abundance [29], albeit potentially suffering from global changes in a near future [30].

Here, using coral reef ecosystems along a geographical gradient, we propose to test two hypotheses. First disparate wilderness areas tend to host a similar level of functional diversity, uncovering a consistent baseline for the breadth of functions in ecosystems, despite a high turnover in species composition. Second, this common level of functional diversity remains highly vulnerable to species declines or losses owing to a disproportional over-redundancy in some functions and a ‘natural’ lack of redundancy for some critical functions.

Coral reefs are the most diverse marine ecosystems on Earth [31] and support key services for half a billion people, such as food and income [32]. We quantified the baseline vulnerability to fishing of fish functional diversity in coral reef ecosystems across the Indo-Pacific geographical gradient, taking advantage of extensive surveys in French Polynesia, New Caledonia and Chagos. These three wilderness areas all benefit from a high level of isolation from humans [33] and a high level of enforcement owing to the presence of military forces, thereby limiting illegal fishing activities. As the ecological knowledge to assess the functions carried by individual species is limited, using species functional traits to infer functions offers a viable alternative [34]. Here, we assume that species with more diverse combinations of functional traits are more likely to support different functions (e.g. [35–37]).

## 2. Material and methods

### (a) Study regions

**Figure 1.**
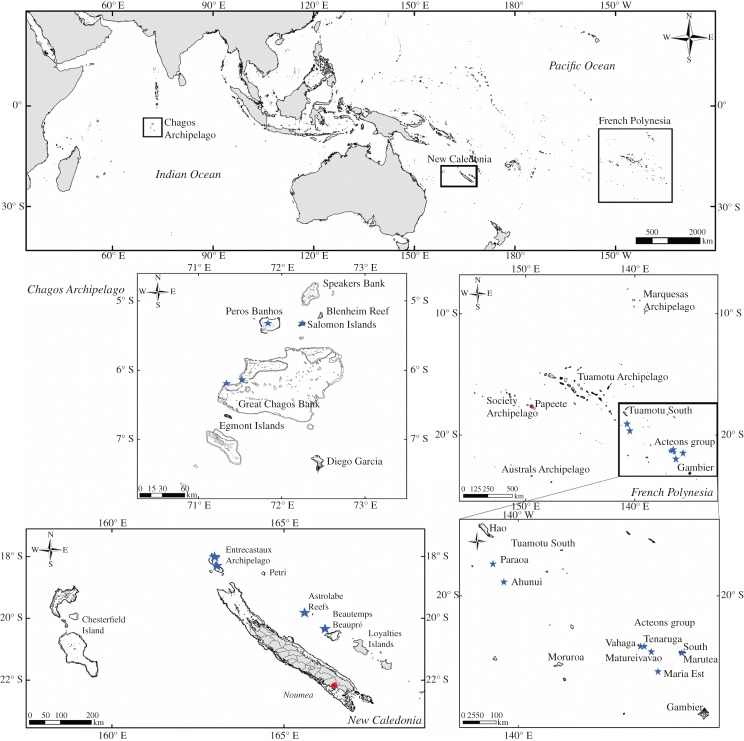
Sampled coral reef ecosystems across three regions of the Indo-Pacific. Fishes were identified and counted at the outer reefs of remote atolls (blue stars) in Chagos (79 transects), New Caledonia (18 transects) and French Polynesia (37 transects).

Remote atolls and islands in three regions (Chagos Archipelago, New Caledonia, and French Polynesia) were sampled along the Indo-Pacific biogeographic gradient, encompassing 130° of longitude (16 000 km) (electronic supplementary material, figure S1). None of these atolls and islands are inhabited: the northern Chagos Archipelago (the Great Chagos Bank, Peros Banhos and Salomon Island) is more than 650 km south of the Maldives and personnel at the Diego Garcia atoll navy base are not permitted to the northern Archipelago other than for fishery patrols; isolated atolls and islands in New Caledonia (Entrecastaux Archipelago, Astrolabe Reef, and Beautemps-Beaupré) are located between 300 and 600 km and more than 20 h by boat from the capital Nouméa [25]; two atolls at the southeast end of the Tuamotu Archipelago (Paraoa and Ahunui) are located approximately 950 km from Papeete, the capital of French Polynesia; and the Acteon Group, a cluster of atolls, is located between 200 and 500 km north of Gambier Island, French Polynesia (figure 1).

### (b) Fish surveys

Fish data for the Pacific Islands were collected on outer reef slopes using Underwater Visual Census (UVC) along 50 m transects. Briefly, this method involved two divers each recording species identity, abundance and body length [38]. Transects in New Caledonia (18 transects) and French Polynesia (37 transects) were truncated at 10 m wide (10 × 50 m strip transects). In the Chagos Archipelago, fishes were surveyed along outer reef slopes using 50 × 5 m strip transects (79 transects). To make surveys comparable among regions, a common area of 500 m^2^ was obtained by randomly aggregating two Chagos transects (250 m^2^). Accumulation curves of species richness were performed for each region to test for potential biases owing to survey techniques (electronic supplementary material, figure S1). Species densities and abundances were estimated for 500 m^2^ transects and averaged for each region. Sharks and rays were removed from the main species list because of their specific traits, some poaching in Chagos, and difficulties in assessing their abundance using UVC [39]. As such, this study focused on 412 fish species belonging to 35 teleost families.

### (c) Functional traits and entities

To estimate functional diversity, we used six functional traits related to major fish attributes: (i) maximum body size, (ii) diet, (iii) home range, (iv) position over the reef, (v) activity, and (vi) gregariousness [21,40].

Fish sizes were coded using six ordered categories: 0–7 cm, 7.1–15 cm, 15.1–30 cm, 30.1–50 cm, 50.1–80 cm and more than 80 cm. Diet was characterized based on the main items consumed by each species, which led to seven trophic categories: herbivorous–detritivorous (i.e. fishes feeding on turf or filamentous algae and/or undefined organic material), macroalgal herbivorous (i.e. fishes eating large fleshy algae and/or seagrass), invertivorous targeting sessile invertebrates (i.e. corals, sponges and ascidians), invertivorous targeting mobile invertebrates (i.e. benthic species such as crustaceans, echinoderms), planktivores (i.e. fishes eating small organisms in the water column), piscivorous (including fishes and cephalopods), and omnivorous (i.e. fishes for which both vegetal and animal material are important in their diet) [21,41]. Home range was coded using three ordered categories: sedentary (including territorial species), mobile within a reef, and mobile between reefs. Position in the water column was coded using three ordered categories: benthic, bentho-pelagic, and pelagic. Activity period was coded using three ordered categories: diurnal, both diurnal and nocturnal, and nocturnal. Schooling was coded using five ordered categories: solitary, pairing or living in small (3–20 individuals), medium (20–50 individuals) or large (more than 50 individuals) groups. This functional trait's database was built using information about the ecology of adult life-stages available in the literature and according to observations made in the Indo-Pacific by the survey team [40,42,43].

More detailed descriptions linking these traits to ecological processes can be found in the electronic supplementary material of published articles [21,28]. Because all traits were coded using categories, we defined functional entities (FEs) as groups of species sharing the same trait categories. In total, 412 fish species were clustered into 157 different FEs [21,28].

### (d) Fish functional diversity

In order to compare the level of functional richness (FRic) among the wilderness areas, we measured the FRic of fishes for each region defined as the volume inside the convex hull occupied by species within a functional space [44,45]. To build a functional space, we calculated pairwise functional distances between species pairs based on the six functional traits using the Gower metric, which allows mixing different types of variables while giving them equal weight [46]. A principal coordinates analysis (PCoA) was performed on this distance matrix to build a multidimensional functional space. We retained the first four principal axes (PCs), which faithfully represent the Gower distance between species [44,45,47].

### (e) Taxonomic and functional *β*-diversity

In order to determine if wilderness areas host different species and functional compositions across the Indo-Pacific, we assessed taxonomic and functional *β*-diversity among regions. We used the *β*-diversity partitioning framework based on the Jaccard dissimilarity index [48,49]. Taxonomic *β*-diversity (*β*_jac_) equals (equation (2.1))2.1
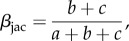
where, *a* is the number of species in both regions A and B, *b* is the number of species present in region A but not in region B and *c* is the number of species present in region B but not in region A.

To distinguish between the species replacement versus nestedness components of *β*-diversity, we decomposed the pairwise Jaccard dissimilarity index (equation (2.1)) into two additive components [48,49]. The replacement component of the Jaccard dissimilarity index (*β*_jtu_, equation (2.2)) describes species replacement without the influence of richness difference between regions. This index is formulated as follows:2.2
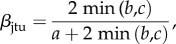
where *a*, *b* and *c* are the same as in Equation 1.

The nestedness component of the Jaccard dissimilarity index (*β*_jne_, equation (2.3)) is the difference between *β*_jac_ and *β*_jtu_. This index accounts for the fraction of dissimilarity owing to richness difference and is formulated as follows:2.3
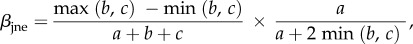
where *a*, *b* and *c* are the same as in equations (2.1) and (2.2). The first term in equation (2.3) expresses a measure of richness difference, whereas the second term corresponds to the dissimilarity version of *β*_jtu_ that is independent of richness difference (1 − *β*_jtu_, [49]).

The functional *β*-diversity among regions was decomposed into functional turnover and functional nestedness-resultant components following the same framework [50,51].

Taxonomic and functional *β*-diversity and their respective components were computed using the R functions from the ‘betapart’ R package (R v. 2.15.2, R development Core Team, 2012).

### (f) Functional vulnerability to fishing

Despite the variety of conceptual approaches, there is growing agreement in defining vulnerability as the combination of three components: (i) *sensitivity*, or the susceptibility of a system to threats, (ii) *exposure*, or the level of those threats on a system, and (iii) *adaptive capacity*, or the capacity of the system to prepare for and respond to those threats [41,52,53]. By analogy, the level of ‘functional vulnerability’ in a given ecosystem relies on (i) *functional sensitivity*, i.e. the extent to which particular traits are more prone to decline in the face of certain threats [54], (ii) *exposure* or the level of threats, and (iii) *functional redundancy*, i.e. the degree to which the same functional traits are supported by many and/or abundant species.

In wilderness areas, exposure to fishing is absent or extremely low [29]. Therefore, the vulnerability to fishing in wilderness areas, termed here as ‘baseline vulnerability’, is solely defined as the combination of species sensitivity to fishing, driven by their biological traits (e.g. size, growth and reproductive capacity), and the level of functional redundancy which is determined by the natural distribution of species density and abundances among FEs (electronic supplementary material, figure S2).

The sensitivity of each fish species to fishing was estimated using a fuzzy logic expert system to take into account eight life-history characteristics that are linked to species productivity and other factors that make fish species more or less sensitive to fishing [55]. This indicator has accurate predictive capacity [55] and has been widely recognized as a comprehensive and suitable indicator of fish sensitivity to fishing [56]. The sensitivity to fishing was aggregated at the level of FEs by averaging the sensitivity of all species belonging to the given FE. The scale ranged from 0 to 100.

Functional redundancy is defined as the level of functional equivalence among species in an ecosystem, such that one function may be performed by one or many species, and one species may substitute for another in the latter case [57]. Here, redundancy was assessed using three complementary indices. First, we used the number of species composing each FE in each region (FR_S_). However, the number of species per FE is only one aspect of functional redundancy. The distribution of species abundances within FEs represents a complementary aspect of redundancy [58–60]. Therefore, secondly we took into account the number of individuals in each FE (FR*_ab_*).

The mean abundance per 500 m^2^ of a species *i* in each region was calculated according to the formula2.4
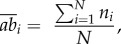
where *N* is the number of transects per region and *n_i_* is the number of individuals of species *i*. Then, functional redundancy of a given FE for a given region FR*_ab_* was obtained as follows:2.5
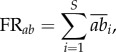
with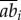 representing the mean number of individuals of species *i* per 500 m^2^ and *S* is the number of species in the given FE for that given region.

Thirdly, we computed a redundancy index accounting for both the number of species and their abundances in each FE. We used the Shannon entropy index FR_Shannon_ with the rationale that a given FE will have more redundancy if represented by many abundant species. Conversely, FEs will have low redundancy if represented by few and rare species. Accordingly, in each region, FR_Shannon_ for each FE was computed as2.6
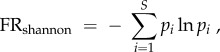
with2.7
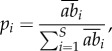
where, *p_i_* is the individuals' proportion of species *i* in the FE, and *S* is the number of species per FE.

We applied the correction derived from the equivalent number of species [61]:2.8
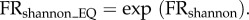
The equivalent number of species is an unbiased measure of Shannon entropy, following Hill's ‘doubling’ property which ensures that the diversity index doubles with the level of diversity, as opposed to nonlinear diversity indices that behave counterintuitively [62].

Quantitatively, the vulnerability of fish FEs to fishing for each region was assessed using a framework based on multi-criteria decision-making (MCDM) and the TOPSIS method (Technique for Order Preference by Similarity to an Ideal Solution). Applied to our specific case, this technique ranks FEs according to their relative distance to the positive and negative ideal solutions, which represent the conditions obtained when the criteria have extreme values [41,63]. The positive ideal solution (A^+^) corresponds to the conditions where sensitivity to threats is minimum while redundancy is maximum (electronic supplementary material, figure S3). Conversely, the negative ideal solution (A^−^) corresponds to the conditions where sensitivity to threats is maximum while redundancy is minimum.

Functional vulnerability was then expressed as the relative distance to these positive and negative ideal solutions according to equation (2.9):2.9
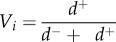
where *V_i_* is the vulnerability of functional entity *i*, *d^+^* is the distance to A^+^ and *d^−^* the distance to A^−^ in Euclidean space. The vulnerability index ranges from 0 if the criteria scores correspond to A^+^ and to 1 when the criteria scores correspond to A^−^ [41,63].

The vulnerability of an FE is high when both the fishing sensitivity of that FE is high and when redundancy is low [41,64].

### (g) Mapping functional sensitivity, redundancy and vulnerability in functional space

The density distribution of functional redundancy, sensitivity and vulnerability within the functional space was estimated using the kernel method with a Gaussian estimation [65]. The smoothing parameter *h* was estimated using the ad hoc method, which is the optimum *h* value obtained for the normal distribution [65]:2.10
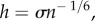
where, *n* is the number of FEs, and *σ*^2^ being the estimated variance for *x* and *y* coordinates:2.11
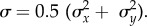
The kernel density estimation was computed using the ‘adehabitatHR’ R package (R v. 2.15.2, R development Core Team, 2014).

Functional redundancy, sensitivity and vulnerability were mapped onto the functional space for each region and their match was estimated with the Pearson product-moment correlation.

## 3. Results

### (a) Similar level of functional diversity across wilderness areas

**Figure 2.**
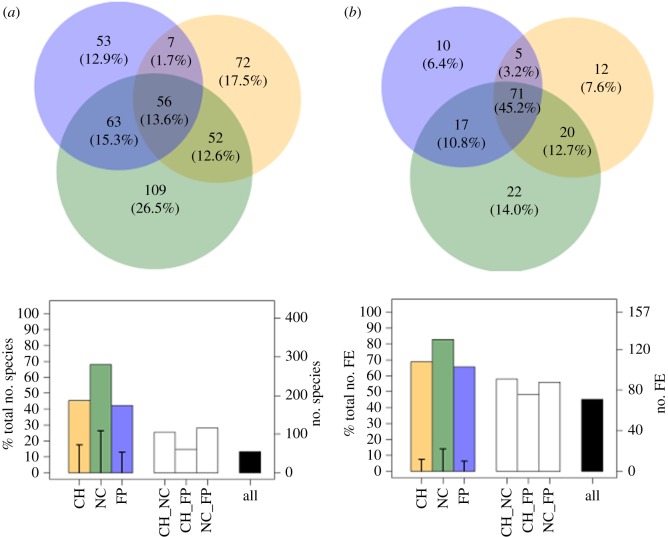
Sharing of species and functional entities between regions. Venn diagram (top) for the number of species (*a*) and the number of functional entities (*b*) for Chagos (orange), New Caledonia (green), and French Polynesia (blue). Percentages indicate the proportion of species and functional entities in each region compared with the total pool. Histograms (bottom) show the proportion (left-axis) and the number of species (*a*) or functional entities (*b*) (right-axis) in each region (colour bars), the proportion of unique species (*a*) or functional entities (*b*) in each region (black segments on coloured bars), the number of species (*a*) or functional entities (*b*) for each pair of regions, and the number of species (*a*) or functional entities (*b*) common to the three regions.

The greatest number of species and FEs was found in New Caledonia, encompassing 83% of FEs recorded in the three regions (figure 2). By contrast, French Polynesia showed the lowest number of species (42%) and FE (69%) (figure 2; additional information in the electronic supplementary material). Overall, 45% of the total FEs (71) were common to the three regions, whereas only 13% (56) of the species were shared (figure 2; additional information in the electronic supplementary material).

The FRic in each region (FRic) ranged from 82% of the global functional volume (French Polynesia) to 94.4% (New Caledonia) (electronic supplementary material, figure S4). The low variability of functional diversity between biogeographic regions is consistent with the weak level of *β*-functional diversity (functions turnover) between regions ranging from 0.14 to 0.18 (maximum is 1). Conversely, the *β*—taxonomic diversity was higher, ranging between 0.66 and 0.79 (maximum at 1) (electronic supplementary material, figure S5, table S1 and additional information), implying that most FEs are present independent of species identities.

### (b) Heterogeneous distribution of functional redundancy across wilderness areas and functional entities

**Figure 3.**
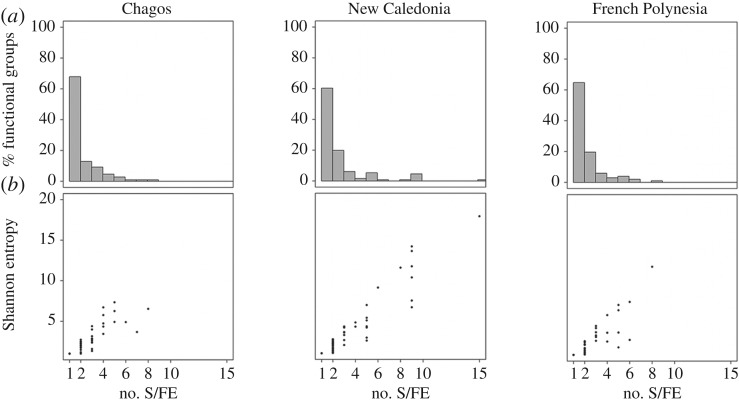
The levels of functional redundancy in terms of species richness and Shannon entropy across functional entities for the three regions. Distribution of functional entities (in percentage) along a gradient of functional redundancy in terms of the number of species by functional entity in each region (*a*). Relationships between the number of species per functional entity and the Shannon entropy (expressed as equivalent number of species, Material and methods) for each functional entity are shown for each region (*b*). The bottom panel demonstrates the variation of Shannon entropy for a fixed number of species per functional entity. no. S/FE is the number of species per functional entity.

Despite the overall stability of functional diversity across the biogeographic gradient, the highest functional redundancy of both species per FE (15) and Shannon entropy (17) was found in New Caledonia, whereas Chagos and French Polynesia only reached 8–9 species (figure 3*b*; additional information in the electronic supplementary material). In each region, more than 60% of FEs were represented by only one species, and 12–20% of the FEs were represented by only two species (figure 3*a*; additional information in the electronic supplementary material), showing that the majority of FEs had low species redundancy.

**Figure 4.**
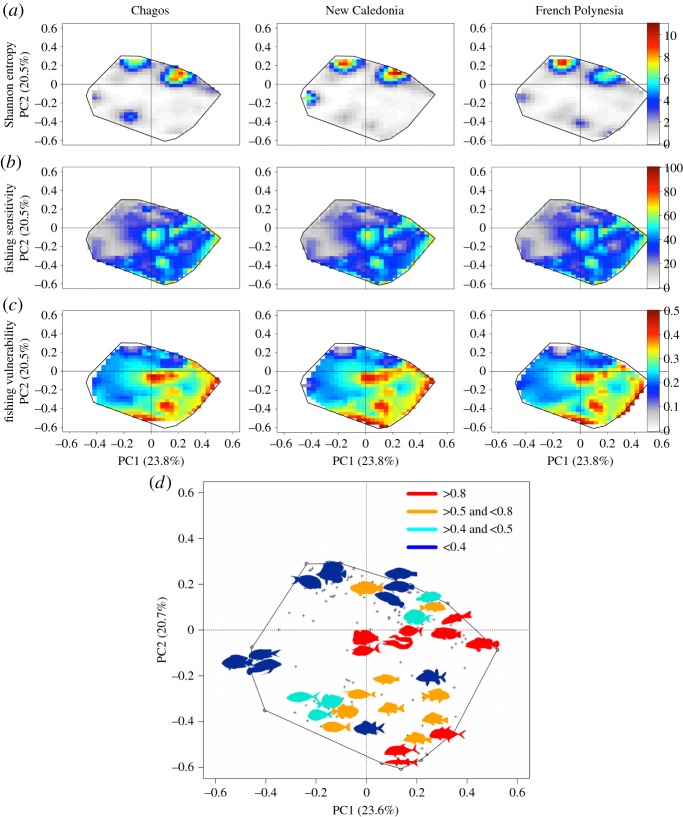
Mapping redundancy, sensitivity and vulnerability in the functional space for fish faunas of the three regions**.** The top row (*a*) shows for each region the distribution of functional redundancy within functional entities, measured using the Shannon entropy, across the functional space. The middle row (*b*) shows the distribution of sensitivity to fishing across the functional space while the bottom row (*c*) shows the distribution of vulnerability to fishing in this space, and (*d*) position of vulnerable functional entities in the functional space. Colours indicate the level of vulnerability of functional entities and fish shapes were chosen to illustrate the main genus of each functional entity (see also the electronic supplementary material, figure S6).

Mapping variation of Shannon entropy across the functional space showed a highly heterogeneous distribution of functional redundancy for each region, with the highest values disproportionally packed into some parts of the functional space (figure 4*a*; electronic supplementary material, figure S7), leaving most of the functional space with low or no redundancy. The extreme concentration of functional redundancy in the top left of the figure (figure 4*a*) was represented by sedentary small to medium size detritivorous, invertivorous, planktivores and omnivorous feeders (e.g. surgeonfishes, damselfishes, butterflyfishes) (figure 4*a*; electronic supplementary material, figure S6). The top right concentration of redundancy (figure 4*a*) was characterized by small to medium size invertivorous feeders (mobile prey) (see also additional information in the electronic supplementary material).

### (c) High vulnerability of functional entities in wilderness areas

As most of the functional space had low functional redundancy, the variation in vulnerability to fishing was partly driven by sensitivity to fishing (mean Pearson's coefficient across regions = 0.93, *p* < 0.001). Variations in functional sensitivity and redundancy across the functional space were independently distributed (mean Pearson's coefficient across locations = −0.09, *p* > 0.1) albeit the most sensitive species systematically showed very low redundancy (electronic supplementary material, figure S8).

Medium to large top predators, both solitary and sedentary, such as groupers (top right) and moray eels (top right), as well as mobile and medium sized schooling species such as the bluefin trevally (*Caranx melampygus*) and the great barracuda (*Sphyraena barracuda*) (bottom right) were highly vulnerable to fishing in the three regions (figure 4*c*,*d*; electronic supplementary material, figure S6) owing to their high sensitivity to fishing (figure 4*b*) and low redundancy (1 species per FE (figure 4*a*)). For the same reasons, large invertivores species feeding on mobile prey such as the humphead wrasse (*Cheilinus undulatus*), large invertivores (sedentary and mobile), detritivores and planktivores, located in the upper right side of the functional space (figure 4*b*; electronic supplementary material, figure S6) showed a high vulnerability to fishing (figure 4*b*; addition information in the electronic supplementary material).

At the centre of the functional space, we observed a high fishing vulnerability peak owing to the presence of two large, mobile and medium sized schooling species; the bumphead parrotfish (*Bolbometopon muricatum*), an invertebrate sessile feeder and the humpback unicornfish (*Naso brachycentron*), a macroalgae feeder (figure 4*b*; electronic supplementary material, figure S6). At the right side of the functional space, we observed a second peak of vulnerability to fishing (hence high sensitivity coupled with low redundancy) characterized by the bicolour parrotfish (*Cetoscarus ocellatus*), a large bioeroder [66] and the bluespine unicornfish (*Naso unicornis*), a macroalgae feeder, both being the only species in their respective FEs and highly sensitive to fishing, explaining their high vulnerability.

Conversely, the high diversity of small to medium size herbivorous–detritivorous, omnivorous, invertivorous and planktivores species in some FEs (top left of the functional space), contributed to their high functional redundancy, counterbalancing (mean Pearson's coefficient across locations of −0.21, *p* < 0.001) their high sensitivity to fishing and making them less vulnerable (figure 4). The higher functional redundancy in New Caledonia decreased the vulnerability of certain FEs. For example, the functional entity composed of medium size invertebrate feeders (mainly from the triggerfishes family) was composed of nine species in New Caledonia and five in French Polynesia, thereby, in concert with Shannon entropy, reducing its vulnerability of 12% compared with French Polynesia. Variation in redundancy and vulnerability across the functional space depicted by the third and fourth axes of PCoA is showed in electronic supplementary material, figure S9.

## 4. Discussion

### (a) Homogeneous level of functional diversity and functional vulnerability across the **I**ndo-**P**acific biogeographic gradient

Surprisingly, despite the large differences in species richness and identity among the three regions, we found very consistent patterns of functional diversity along the species richness biogeographic gradient [67], suggesting that the set of ecological functions for the growth and persistence of coral reefs are supported independently of species identity and richness, thus serving as a ‘natural’ baseline indicator. As such, the presence/absence of FEs compared with wilderness areas may be a useful indicator to assess ecosystem conditions under human pressure. This result is in line with a previous study on the scale of biogeographic regions demonstrating that functions are maintained along a gradient of species richness [21]. Similar consistency was found for functional vulnerability, suggesting that combinations of fish traits have quasi-similar levels of redundancy across coral reefs and the biogeographic gradient, albeit New Caledonia had lower levels of vulnerability for several FEs owing to higher functional redundancy.

### (b) High vulnerability of ecosystem functioning

Studies of vulnerability have typically focused on species rather than ecological functions [41], and often overlook abundance distributions among species [21,41]. In addition, the assumption that functional diversity in wilderness areas, seen as a reference, is not buffered against extinction owing to relatively high functional vulnerability has never been tested. Here, we filled this gap by assessing the baseline functional vulnerability of fish assemblages to fishing in some of the most isolated coral reef ecosystems across the Indo-Pacific. We found that even in the near-absence of fishing and considering species abundance, most FEs remain highly vulnerable in each wilderness ecosystem because species diversity is overwhelmingly packed into a small set of FEs, leaving most FEs with no redundancy or functional insurance. Importantly, functional sensitivity and functional redundancy are independently distributed among the combinations of traits. As such, redundancy is seldom compensating for the high sensitivity of some traits, the most sensitive ones often having no redundancy.

Our study is complementary to previous macro-ecological investigations showing the heterogeneous distribution of redundancy across FEs [21,41], and attests that the same pattern translates to the local scale when considering species abundances. This result is not trivial because even FEs with low species redundancy may be preserved if those species are widespread, abundant and weakly sensitive to threats. This is not the case for fishes on coral reefs. Consequently, high biodiversity and abundance of fishes is unlikely to buffer coral reef ecosystems against functional diversity loss. This result is consistent with the observations that marine ecosystem functions and services scale positively and do not saturate with the level of species and FE richness within local faunas [68,69].

Vulnerability can provide early warning signals of fishing pressure, because the processes performed by vulnerable traits are most likely to be the first to decline, even under low pressure [70]. Indeed, from a sustainable management perspective, this study highlights key vulnerable FEs that should be under particular conservation measures. For example, the bluespine unicornfish (*N. unicornis*) has been identified as one of the most important macroalgal consumers on coral reefs [71] yet is targeted by commercial, recreational and artisanal fisheries [72]. Macroalgal feeders play a critical role in preventing and reversing coral-algal shifts and the finding of their high baseline vulnerability to fishing is worrying [8,9]. Despite recent studies demonstrating signs of overexploitation in the Pacific [72], this species is listed as ‘Least concern’ in the Red list of Threatened Species by the International Union for Conservation of Nature (IUCN). This is also the case for top predators such as the bluefin trevally (*C. melampygus*) and the great barracuda (*S. barracuda*) which are highly sensitive to fishing [73,74], while the bluefin trevally has also been found in very low abundance in marine PAs compared with wilderness [28]. The IUCN Red list criteria could benefit from taking a trait based approach to uncover the species vulnerability from a functional perspective.

## 5. Conclusion

With biodiversity being lost in the Anthropocene, the need to carefully manage functional diversity has never been greater. The slow decrease in abundance of functionally important species is insidious, despite strong implications for coral ecosystem functioning and resilience [9]. High redundancy can highlight the core processes that may persist even under high levels of fishing pressure because they are highly buffered against local species extinction. While fishing pressure should decrease on the most vulnerable FEs, it could potentially be directed toward the least vulnerable FEs highlighted in the functional space. Local adaptive management measures toward those species could focus on gear restrictions, minimum size limits [75], quotas or fishing bans during spawning times [76,77] or modified angling techniques (e.g. [78–80]). However, those species often do not garner the same fishing interest owing to their low commercial values [81]. Therefore, the burden of reducing fishing pressure on the most vulnerable FEs should be shared along market value chains [82], as fisherman targeting certain species operate under incentives determined by trade and the agents involved at both local and international scales [83,84]. Ultimately, a multilayer management approach considering the complexity of the socio-ecological system is necessary to maintain ecosystem functioning [82].

## Supplementary Material

Additional information

## Supplementary Material

Supplemental Figures and Tables

## Data accessibility

Functional entity, fishing sensitivity and vulnerability on Dryad: http://dx.doi.org/10.5061/dryad.24gs0 [85].

## References

[1] Vitousek PM 1997 Human domination of Earth's ecosystems. Science 277, 494–499. (10.1126/science.277.5325.494)

[2] Jackson JB et al. 2001 Historical overfishing and the recent collapse of coastal ecosystems. Science 293, 629–637. (10.1126/science.1059199) 11474098

[3] Waters CN et al. 2016 The Anthropocene is functionally and stratigraphically distinct from the Holocene. Science 351, 137–147. (10.1126/science.aad2622) 26744408

[4] Costello MJ, May RM, Stork NE 2013 Can we name Earth's species before they go extinct? Science 339, 413–416. (10.1126/science.1230318) 23349283

[5] Worm B et al. 2006 Impacts of biodiversity loss on ocean ecosystem services. Science 314, 787–790. (10.1126/science.1132294) 17082450

[6] Barnosky AD et al. 2012 Approaching a state shift in Earth's biosphere. Nature 486, 52–58. (10.1038/nature11018) 22678279

[7] Pimm SL, Jenkins CN, Abell R, Brooks TM, Gittleman JL, Joppa LN, Ravan PH, Roberts CM, Sexton JO 2014 The biodiversity of species and their rates of extinction, distribution, and protection. Science 344, 1246752 (10.1126/science.1246752) 24876501

[8] Hughes TP et al. 2007 Phase shifts, herbivory, and the resilience of coral reefs to climate change. Curr. Biol. 17, 360–365. (10.1016/j.cub.2006.12.049) 17291763

[9] Bellwood DR, Hughes TP, Folke C, Nyström M 2004 Confronting the coral reef crisis. Nature 429, 827–833. (10.1038/nature02691) 15215854

[10] Naeem S 2012 Ecological consequences of declining biodiversity: a biodiversity-ecosystem function (BEF) framework for marine systems. In Marine biodiversity and ecosystem functioning: frame-works, methodologies and integration (eds M Solan *et al*.), pp. 34–51. Oxford, UK: Oxford University Press.

[11] Hooper DU et al. 2012 A global synthesis reveals biodiversity loss as a major driver of ecosystem change. Nature 486, 105–108. (10.1038/nature11118) 22678289

[12] Seddon N, Mace G, Naeem S et al. 2016 Biodiversity in the Anthropocene: prospects and policy. Proc. R. Soc. B 283. 10.1098/rspb.2016.2094 PMC5204156 27928040

[13] Lefcheck JS et al. 2015 Biodiversity enhances ecosystem multifunctionality across trophic levels and habitats. Nat. Commun. 6, 6936 (10.1038/ncomms7936) 25907115 PMC4423209

[14] Gascon C et al. 2015 The importance and benefits of species. Curr. Biol. 25, R431–R438. (10.1016/j.cub.2015.03.041) 25989087

[15] Duffy JE, Lefcheck JS, Stuart-Smith RD, Navarrete SA, Edgar GJ 2016 Biodiversity enhances reef fish biomass and resistance to climate change. Proc. Natl Acad. Sci. USA 113, 6230–6235. (10.1073/pnas.1524465113) 27185921 PMC4896699

[16] Norkko A, Villnäs A, Norkko J, Valanko S, Pilditch C 2013 Size matters: implications of the loss of large individuals for ecosystem function. Sci. Rep. 3, 2646 (10.1038/srep02646) 24025973 PMC6505624

[17] Tilman D, Knops J, Wedin D, Reich P, Ritchie M, Siemann E 1997 The influence of functional diversity and composition on ecosystem processes. Science (80-) 277, 1300–1302. (10.1126/science.277.5330.1300)

[18] Naeem S, Li S 1997 Biodiversity enhances ecosystem reliability. Nature 390, 507–509. (10.1038/37348)

[19] Yachi S, Loreau M 1999 Biodiversity and ecosystem productivity in a fluctuating environment: the insurance hypothesis. Proc. Natl Acad. Sci. USA 96, 1463–1468. (10.1073/pnas.96.4.1463) 9990046 PMC15485

[20] Isbell F et al. 2015 Biodiversity increases the resistance of ecosystem productivity to climate extremes. Nature 526, 574–577. (10.1038/nature15374) 26466564

[21] Mouillot D et al. 2014 Functional over-redundancy and high functional vulnerability in global fish faunas on tropical reefs. Proc. Natl Acad. Sci. USA 111,13 757–13 762. (10.1073/pnas.1317625111) PMC4183327 25225388

[22] Mouillot D et al. 2013 Rare species support vulnerable functions in high- diversity ecosystems. PLoS Biol. 11, e1001569 (10.1371/journal.pbio.1001569) 23723735 PMC3665844

[23] Soliveres S et al. 2016 Biodiversity at multiple trophic levels is needed for ecosystem multifunctionality. Nature 536, 456–459. (10.1038/nature19092) 27533038

[24] Edgar GJ et al. 2014 Global conservation outcomes depend on marine protected areas with five key features. Nature 506, 216–220 (10.1038/nature13022) 24499817

[25] Knowlton N, Jackson JBC 2008 Shifting baselines, local impacts, and global change on coral reefs. PLoS Biol. 6, e54 (10.1371/journal.pbio.0060054) 18303956 PMC2253644

[26] McClanahan TR, Omukoto JO 2011 Comparison of modern and historical fish catches (AD 750–1400) to inform goals for marine protected areas and sustainable fisheries. Conserv. Biol. 25, 945–955. (10.1111/j.1523-1739.2011.01694.x) 21676028

[27] Kathleen Lyons S et al. 2015 Holocene shifts in the assembly of plant and animal communities implicate human impacts. Nature 529, 80–83. (10.1038/nature16447) 26675730

[28] D'agata S, Mouillot D, Wantiez L, Friedlander AM, Kulbicki M, Vigliola L 2016 Marine reserves lag behind wilderness in the conservation of key functional roles. Nat. Commun. 7, 12000 (10.1038/ncomms12000) 27354026 PMC4931279

[29] Mittermeier RA, Mittermeier CG, Brooks TM, Pilgrim JD, Konstant WR, da Fonseca GAB, Kormos C 2003 Wilderness and biodiversity conservation. Proc. Natl Acad. Sci. USA 100, 10 309–10 313. (10.1073/pnas.1732458100) 12930898 PMC193557

[30] Watson JEM, Shanahan DF, Di Marco M, Allan J, Laurance WF, Sanderson EW, Mackey B, Venter O 2016 Catastrophic declines in wilderness areas undermine global environment targets. Curr. Biol. 26, 2929–2934. (10.1016/j.cub.2016.08.049) 27618267

[31] Spalding MD, Ravilious C, Green EP 2001 Word atlas of coral reefs. Berkeley, CA: University of California Press, prepared at the UNEP World Conservation Monitoring Centre.

[32] Cinner JE 2014 Coral reef livelihoods. Curr. Opin. Environ. Sustain. 7, 65–71. (10.1016/j.cosust.2013.11.025)

[33] Maire E et al. 2016 How accessible are coral reefs to people? A global assessment based on travel time. Ecol. Lett. 19, 351–364. (10.1111/ele.12577) 26879898

[34] Violle C, Navas M, Vile D, Kazakou E, Fortunel C, Hummel I, Garnier E 2007 Let the concept of trait be functional ! Oikos 116, 882–892. (10.1111/j.2007.0030-1299.15559.x)

[35] Coux C, Rader R, Bartomeus I, Tylianakis JM 2016 Linking species functional roles to their network roles. Ecol. Lett. 19, 762–770. (10.1111/ele.12612) 27169359

[36] Murray F, Douglas A, Solan M 2014 Species that share traits do not necessarily form distinct and universally applicable functional effect groups. Mar. Ecol. Prog. Ser. 516, 23–34. (10.3354/meps11020)

[37] Pigot A, Bregman T, Sheard C et al. 2016 Quantifying species contributions to ecosystem process: a global assessment of functional trait and phylogenetic metrics across seed-dispersal network. Proc. R. Soc. B 283. 10.1098/rspb.2016.1597 PMC5204145 27928035

[38] Labrosse P, Kulbicki M, Ferraris J 2002 *Underwater visual fish census surveys. Proper use and implementation*. Reef Resour Assess tools Noumea, New Caledonia Secr Pacific Community 54 p.

[39] Ward-Paige C, Mills Flemming J, Lotze HK 2010 Overestimating fish counts by non-instantaneous visual censuses: consequences for population and community descriptions. PLoS ONE 5, e11722 (10.1371/journal.pone.0011722) 20661304 PMC2908695

[40] Kulbicki M, Mou-tham G, Vigliola L et al. 2011 Major coral reef fish species of the South pacific with basic information on their biology and ecology. Cris. report. Noumea, IRD. 107 pp. + Annex.

[41] Parravicini V et al. 2014 Global mismatch between species richness and vulnerability of reef fish assemblages. Ecol. Lett. 17, 1101–1110. (10.1111/ele.12316) 24985880

[42] Kulbicki M, Bozec Y, Labrosse P, Letourneur Y, Mou-Tham G, Wantiez L 2005 Diet composition of carnivorous fishes from coral reef lagoons of New Caledonia. Aquat. Living Resour. 18, 231–250. (10.1051/alr:2005029)

[43] Chabanet P, Guillemot N, Kulbicki M, Vigliola L, Sarramegna S 2010 Baseline study of the spatio-temporal patterns of reef fish assemblages prior to a major mining project in New Caledonia (South Pacific). Mar. Pollut. Bull. 61, 598–611. (10.1016/j.marpolbul.2010.06.032) 20637479

[44] Villéger S, Mason NWH, Mouillot D 2008 New multidimensional functional diversity indices for a multifaceted framework in functional ecology. Ecology 89, 2290–2301. (10.1890/07-1206.1) 18724739

[45] Mouillot D, Graham NAJ, Villéger S, Mason NWH, Bellwood DR 2013 A functional approach reveals community responses to disturbances. Trends Ecol. Evol. 28, 167–177. (10.1016/j.tree.2012.10.004) 23141923

[46] Legendre P, Legendre L 1998 Numerical ecology. Developmental environment modelling. Amsterdam, The Netherlands: Elsevier Science.

[47] Maire E, Grenouillet G, Brosse S, Villéger S 2015 How many dimensions are needed to accurately assess functional diversity? A pragmatic approach for assessing the quality of functional spaces. Glob. Ecol. Biogeogr. 24, 728–740. (10.1111/geb.12299)

[48] Baselga A 2012 The relationship between species replacement, dissimilarity derived from nestedness, and nestedness. Glob. Ecol. Biogeogr. 21, 1223–1232. (10.1111/j.1466-8238.2011.00756.x)

[49] Baselga A 2010 Partitioning the turnover and nestedness components of beta diversity. Glob. Ecol. Biogeogr. 19, 134–143. (10.1111/j.1466-8238.2009.00490.x)

[50] Villéger S, Grenouillet G, Brosse S 2013 Decomposing functional β-diversity reveals that low functional β-diversity is driven by low functional turnover in European fish assemblages. Glob. Ecol. Biogeogr. 22, 671–681. (10.1111/geb.12021)

[51] Villéger S, Novack-Gottshall PM, Mouillot D 2011 The multidimensionality of the niche reveals functional diversity changes in benthic marine biotas across geological time. Ecol. Lett. 14, 561–568. (10.1111/j.1461-0248.2011.01618.x) 21481126

[52] Foden WB et al. 2013 Identifying the world's most climate change vulnerable species: a systematic trait-based assessment of all birds, amphibians and corals. PLoS ONE 8, e65427 (10.1371/journal.pone.0065427) 23950785 PMC3680427

[53] Cinner JE, McClanahan TR, Graham NAJ, Daw TM, Maina J, Stead SM, Wamukota A, Brown K, Bodin Ö 2012 Vulnerability of coastal communities to key impacts of climate change on coral reef fisheries. Glob. Environ. Change 22, 12–20. (10.1016/j.gloenvcha.2011.09.018)

[54] Graham NAJ et al. 2011 Extinction vulnerability of coral reef fishes. Ecol. Lett. 14, 341–348. (10.1111/j.1461-0248.2011.01592.x) 21320260 PMC3627313

[55] Cheung WWL, Pitcher TJ, Pauly D 2005 A fuzzy logic expert system to estimate intrinsic extinction vulnerabilities of marine fishes to fishing. Biol. Conserv. 124, 97–111. (10.1016/j.biocon.2005.01.017)

[56] Reynolds JD, Dulvy NK, Goodwin NB, Hutchings JA 2005 Biology of extinction risk in marine fishes. Proc. R. Soc. B 272, 2337–2344. (10.1098/rspb.2005.3281) PMC1559959 16243696

[57] Naeem S 1998 Species redundancy and ecosystem reliability. Conserv. Biol. 12, 39–45. (10.1046/j.1523-1739.1998.96379.x)

[58] Bellwood DR, Hughes TP, Hoey AS 2006 Sleeping functional group drives coral-reef recovery. Curr. Biol. 16, 2434–2439. (10.1016/j.cub.2006.10.030) 17174918

[59] Stevens RD, Cox SB, Strauss RE, Willig MR 2003 Patterns of functional diversity across an extensive environmental gradient: vertebrate consumers, hidden treatments and latitudinal trends. Ecol. Lett. 6, 1099–1108. (10.1046/j.1461-0248.2003.00541.x)

[60] Micheli F, Halpern BS 2005 Low functional redundancy in coastal marine assemblages. Ecol. Lett. 8, 391–400. (10.1111/j.1461-0248.2005.00731.x)

[61] Jost L 2006 Entropy and diversity. Oikos 113, 363–375. (10.1111/j.2006.0030-1299.14714.x)

[62] Hill MO 1973 Diversity and evenness: a unifying notation and its consequences. Ecology 54, 427–432. (10.2307/1934352)

[63] Turner BL et al. 2003 A framework for vulnerability analysis in sustainability science. Proc. Natl. Acad. Sci. USA 100, 8074–8079. (10.1073/pnas.1231335100) 12792023 PMC166184

[64] Cinner JE, Huchery C, Darling ES, Humphries AT, Graham NAJ, Hicks CC, Marshall N, McClanahan TR 2013 Evaluating social and ecological vulnerability of coral reef fisheries to climate change. PLoS ONE 8, e74321 (10.1371/journal.pone.0074321) 24040228 PMC3770588

[65] Worton BJ 1995 Using Monte Carlo simulation to evaluate kernel-based home range estimators. J. Wildl. Manage. 59, 794–800. (10.2307/3801959)

[66] Green AL, Bellwood DR 2009 Monitoring functional groups of herbivorous reef fishes as indicators of coral reef resilience: a practical guide for coral reef managers in the Asia Pacific region. Gland, Switzerland: IUCN.

[67] Allen GR 2008 Conservation hotspots of biodiversity and endemism for Indo-Pacific coral reef fishes. Aquat. Conserv. Mar. Freshw. Ecosyst. 556, 541–556. (10.1002/aqc.880)

[68] Danovaro R, Gambi C, Dell'Anno A, Corinaldesi C, Fraschetti S, Vanreusel A, Vincx M, Gooday AJ 2008 Exponential decline of deep-sea ecosystem functioning linked to benthic biodiversity loss. Curr. Biol. 18, 1–8. (10.1016/j.cub.2007.11.056) 18164201

[69] Mora C et al. 2011 Global human footprint on the linkage between biodiversity and ecosystem functioning in reef fishes. PLoS Biol. 9, e1000606 (10.1371/journal.pbio.1000606) 21483714 PMC3071368

[70] Bellwood DR, Hoey AS, Hughes TP 2012 Human activity selectively impacts the ecosystem roles of parrotfishes on coral reefs. Proc. R. Soc. B 279, 1621–1629. (10.1098/rspb.2011.1906) PMC3282342 22090383

[71] Hoey AS, Bellwood DR 2009 Limited functional redundancy in a high diversity system: single species dominates key ecological process on coral reefs. Ecosystems 12, 1316–1328. (10.1007/s10021-009-9291-z)

[72] Ford AK, Bejarano S, Marshell A, Mumby PJ 2016 Linking the biology and ecology of key herbivorous unicornfish to fisheries management in the Pacific. Aquat. Conserv. Mar. Freshw. Ecosyst. 26, 790–805. (10.1002/aqc.2623)

[73] Friedlander A, DeMartini E 2002 Contrasts in density, size, and biomass of reef fishes between the northwestern and the main Hawaiian islands: the effects of fishing down apex predators. Mar. Ecol. Prog. Ser. 230, 253–264. (10.3354/meps230253)

[74] Meyer CG, Holland KN, Wetherbee BM, Lowe CG 2001 Diet, resource partitioning and gear vulnerability of Hawaiian jacks captured in fishing tournaments. Fish. Res. 53, 105–113. (10.1016/S0165-7836(00)00285-X)

[75] Bozec Y-M, O'Farrell S, Bruggemann JH, Luckhurst BE, Mumby PJ 2016 Tradeoffs between fisheries harvest and the resilience of coral reefs. Proc. Natl Acad. Sci. USA 113, 4536–4541. (10.1073/pnas.1601529113) 27044106 PMC4843468

[76] Bejarano Chavarro S, Mumby PJ, Golbuu Y 2014 Changes in the spear fishery of herbivores associated with closed grouper season in Palau, Micronesia. Anim. Conserv. 17, 133–143. (10.1111/acv.12066)

[77] Cohen PJ, Alexander TJ 2013 Catch rates, composition and fish size from reefs managed with periodically-harvested closures. PLoS ONE 8, e73383 (10.1371/journal.pone.0073383) 24066044 PMC3774770

[78] Hicks CC, McClanahan TR 2012 Assessing gear modifications needed to optimize yields in a heavily exploited, multi-species, seagrass and coral reef fishery. PLoS ONE 7, e36022 (10.1371/journal.pone.0036022) 22574133 PMC3344850

[79] Mbaru EK, McClanahan TR 2013 Escape gaps in African basket traps reduce bycatch while increasing body sizes and incomes in a heavily fished reef lagoon. Fish. Res. 148, 90–99. (10.1016/j.fishres.2013.08.011)

[80] McClanahan TR, Sebastian CR, Cinner J et al. 2010 Managing fishing gear to encourage ecosystem-based management of coral reefs fisheries. In *Proc. 11th Int Coral Reef Symp Ft Lauderdale*, Florida 2, 10190–1023 (Session number 22).

[81] Thyresson M, Crona B, Nyström M, de la Torre-Castro M, Jiddawi N 2013 Tracing value chains to understand effects of trade on coral reef fish in Zanzibar, Tanzania. Mar. Policy 38, 246–256. (10.1016/j.marpol.2012.05.041)

[82] McClanahan TR, Cinner JE 2012 Adapting to a changing environment: confronting the consequences of climate change. Oxford, UK: Oxford University Press.

[83] Thyresson M, Nyström M, Crona B 2011 Trading with resilience: parrotfish trade and the exploitation of key-ecosystem processes in coral reefs. Coast Manage. 39, 396–411. (10.1080/08920753.2011.589226)

[84] Williams ID, White DJ, Sparks RT, Lino KC, Zamzow JP, Kelly ELA, Ramey HL 2016 Responses of herbivorous fishes and benthos to 6 years of protection at the Kahekili Herbivore Fisheries Management Area, Maui. PLoS ONE 11, e0159100 (10.1371/journal.pone.0159100) 27462981 PMC4963024

[85] D'agata S et al. 2016 Data from: Unexpected high vulnerability of functions in wilderness areas: evidence from coral reef fishes. Dryad Digital Repository. (10.5061/dryad.24gs0) PMC5204136 27928042

