# Supplementary material for: Unexpected high vulnerability of functions in wilderness areas: evidence from coral reef fishes
Source: Proc Biol Sci. 2016 Dec 14;283(1844):20160128. doi: 10.1098/rspb.2016.0128 (PMC5204136; doi:10.1098/rspb.2016.0128)
Supplement: Additional information [file rspb20160128supp1.pdf]

Electronic Supplementary Material (ESM)

Additional information

Taxonomic and functional diversity of fish assemblages within and across regions

New Caledonia hosted both the highest number of unique species (109, 26.5% of the total species pool) and the highest number of unique functional entities (22, 14.0% of the total functional entities) whereas French Polynesia had the lowest number of unique species (53, 12.4 of the total species pool) and the lowest number of unique functional entities (10, 6.4% of the total functional entities) (Figure 2).

The highest levels of taxonomic β-nestedness (0.15, 23% of the total taxonomic β-diversity) and functional β-nestedness (0.13, 71% of the total functional β-diversity) occurred between New Caledonia and French Polynesia demonstrating that functional diversity in French Polynesia is a subset of that observed in New Caledonia (ESM, Figure S5) while partially sharing taxonomic diversity. In contrast, the lowest taxonomic and functional β -nestedness occurred between Chagos and French Polynesia (0.009 - 1% of the total taxonomic β–diversity and 0.048 - 31% of the total functional β–diversity, respectively), indicating that the differences in taxonomic and functional diversity between these two regions are due to true replacement (turnover) of species and FEs (ESM, Figure S5).

Functional sensitivity peaks

Also, large invertivorous species feeding on mobile prey such as large wrasses (*Cheilinus undulatus* and *Coris*), large pufferfishes (*Arothron*), triggerfishes (*Balistoides, and Pseudobalistes*) and grunts (*Plecthorinchus)* located in the upper right side of the functional space showed a high sensitivity to fishing (Figure 3b).

Functional redundancy peaks

On average, the Shannon entropy index reached 1.82 equivalent numbers of species (sd: 2.26) and ranged from 1 to 18 for the three regions, with the maximum being observed in New Caledonia. This index showed large variations among FEs despite a similar number of species, suggesting that beyond the number of species, the distribution of abundance is also key to functional redundancy (Figure 4). For example in New Caledonia, the Shannon entropy for FEs with 9 species ranged from 7 to 14, implying that FEs with the lowest values are dominated (in abundance) by few species, and inversely for FEs with the highest Shannon entropy values.

Mapping of functional redundancy in the functional space

The top right second peak of redundancy (Figure 3a) was characterized by small to medium size invertivorous feeders (mobile preys) belonging to genera such as *Cheilinus*, *Anampses*, *Bodianus*, *Halichoeres* (wrasses),

Plecthorinchus (grunts), and *Balistapus* (triggerfishes) as well as medium size omnivorous feeders of genus *Scarus* (parrotfishes) (Figure 3a & ESM Figure S6).

Another functional redundancy peak, more pronounced in New Caledonia, was located at the left extreme side of the functional space (Figure 3a) and was characterized by small, sedentary, and large schools of planktivores species belonging mainly to the damselfish family (*Chromis*, *Pomacentrus*, *Amblyglyphidodon*), the dartfishs and one genus of wrasse (*Cirrhilabrus*) (Figure 3a & ESM Figure S6). A high diversity of species per FE was observed in Chagos with medium size, large schooling planktivores species of the fusiliers family (bottom left) (Figure 3a & ESM Figure S6).

Functional vulnerability peaks

Overall, the vulnerability of FEs decreased with increasing species diversity (mean Pearson coefficient across regions of -0.27). The most vulnerable FEs ($V > 0.8$) were all represented by only one species, whereas the least vulnerable FEs ($V < 0.4$) tended to be the most species rich FEs, with the largest values of Shannon entropy (ESM, Figure S9).
