# Supplementary material for: Unexpected high vulnerability of functions in wilderness areas: evidence from coral reef fishes
Source: Proc Biol Sci. 2016 Dec 14;283(1844):20160128. doi: 10.1098/rspb.2016.0128 (PMC5204136; doi:10.1098/rspb.2016.0128)
Supplement: Supplemental Figures and Tables [file rspb20160128supp2.pdf]

**Electronic Supplementary Material (ESM)**

**Figures and Tables**

# Unexpected high vulnerability of functions in wilderness areas: evidence from coral reef fishes

Stéphanie D'agata[1,2,3], Laurent Vigliola[2], Nicholas A.J. Graham[4,5], Laurent Wantiez[6], Valeriano Parravicini[7], Sébastien Villéger[1], Gerard Mou-Tham[2], Philippe Frolla[8], Alan M. Friedlander[9,10], Michel Kulbicki[11] & David Mouillot[1,4]

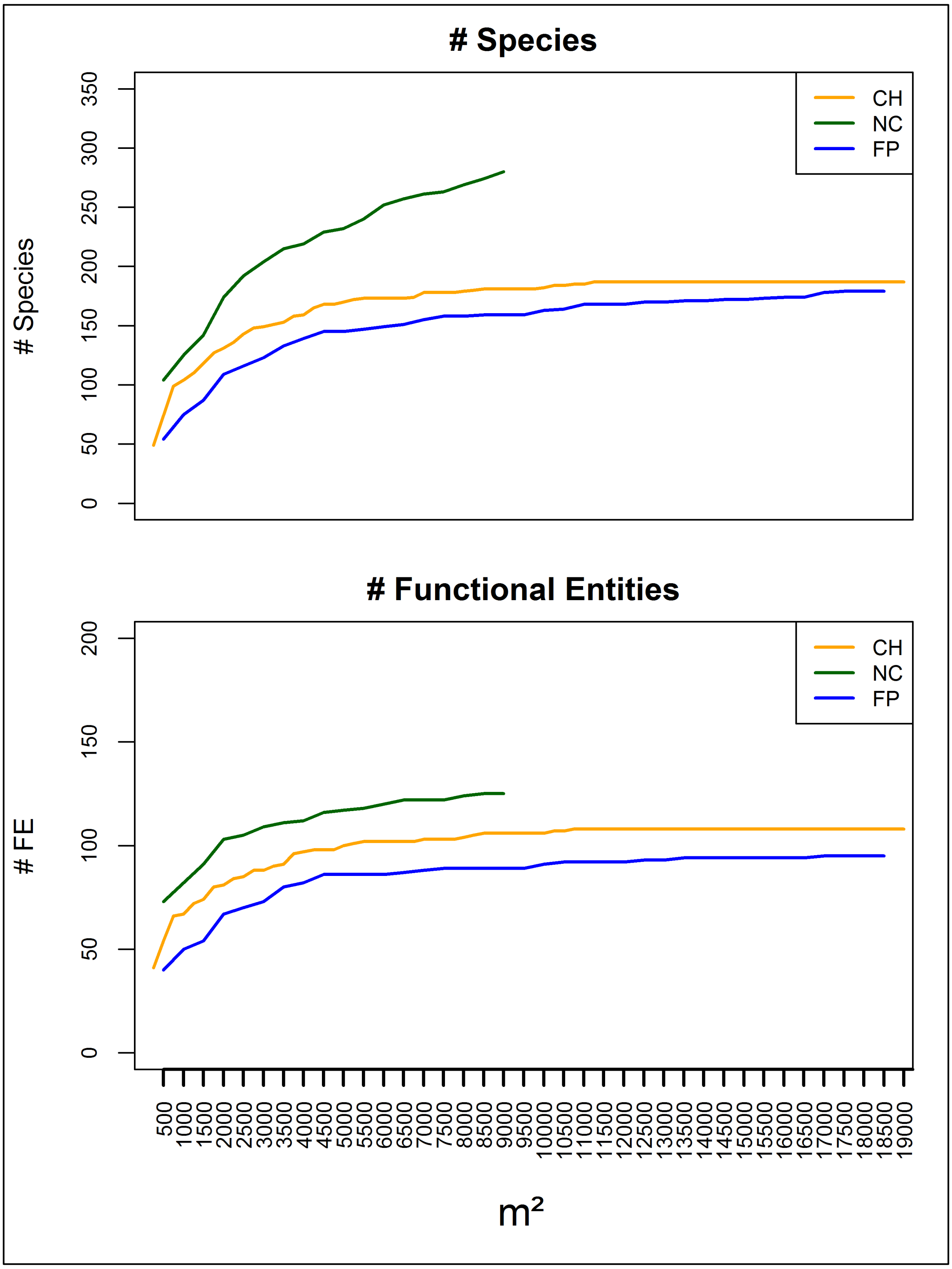


**Figure S1**. **Accumulation curves for species richness and functional entities**
Accumulation curves for the fish richness in terms species (top) and functional entities per area (bottom) for each of the three regions (Chagos (CH), orange; New Caledonia (NC), Green; French Polynesia (FP), blue) by cumulating transects (x-axis, m²).

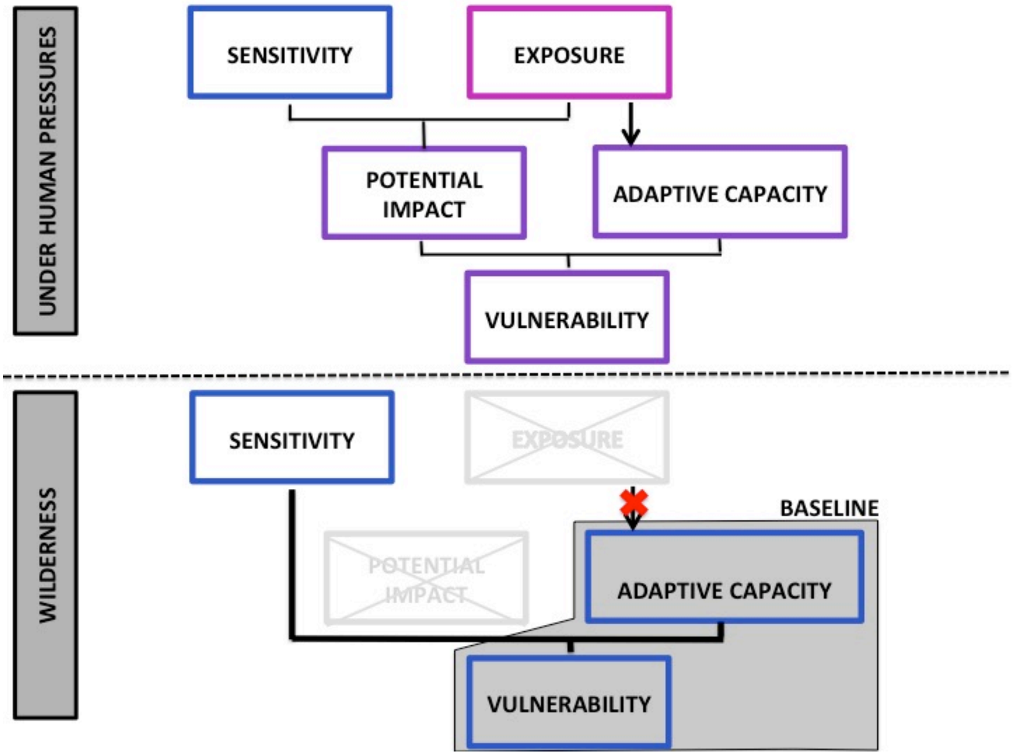


**Figure S2. Conceptual framework for assessing the level of natural functional vulnerability in ecosystems.** Sensitivity of a species refers to its susceptibility to be affected by a given threat. Sensitivity coupled with exposure to external threat (pink) determines potential impacts. Actual vulnerability of a system to disturbance results from the effect of adaptive capacity. In wilderness areas, vulnerability to fishing is only function of sensitivity and functional redundancy since the level of exposure is reduced to its minimum due to the quasi absence of direct human impacts.

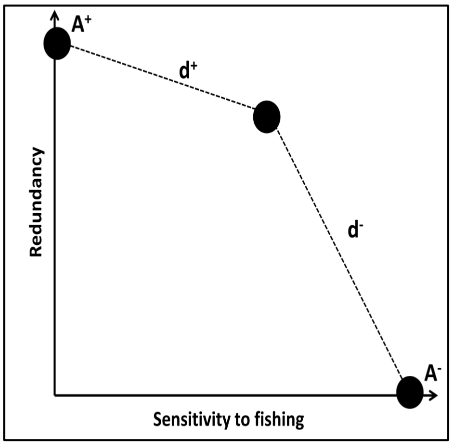


**Figure S3: Conceptual plot exemplifying the multicriteria method employed for the quantification of vulnerability based on the sensitivity to fishing and redundancy within reef fish assemblages.**

The vulnerability of the focal functional entity is quantified as its relative distance to the positive ideal solution $A^+$ and the negative ideal solution $A^-$, where $A^+$ represents an ideal assemblage with minimum vulnerability (i.e. minimum sensitivity to fishing and maximum redundancy) and $A^-$ represents a full negative scenario with maximum sensitivity to fishing and minimum redundancy. Vulnerability (V) is then numerically expressed as $V = d^+ / (d^+ + d^-)$.

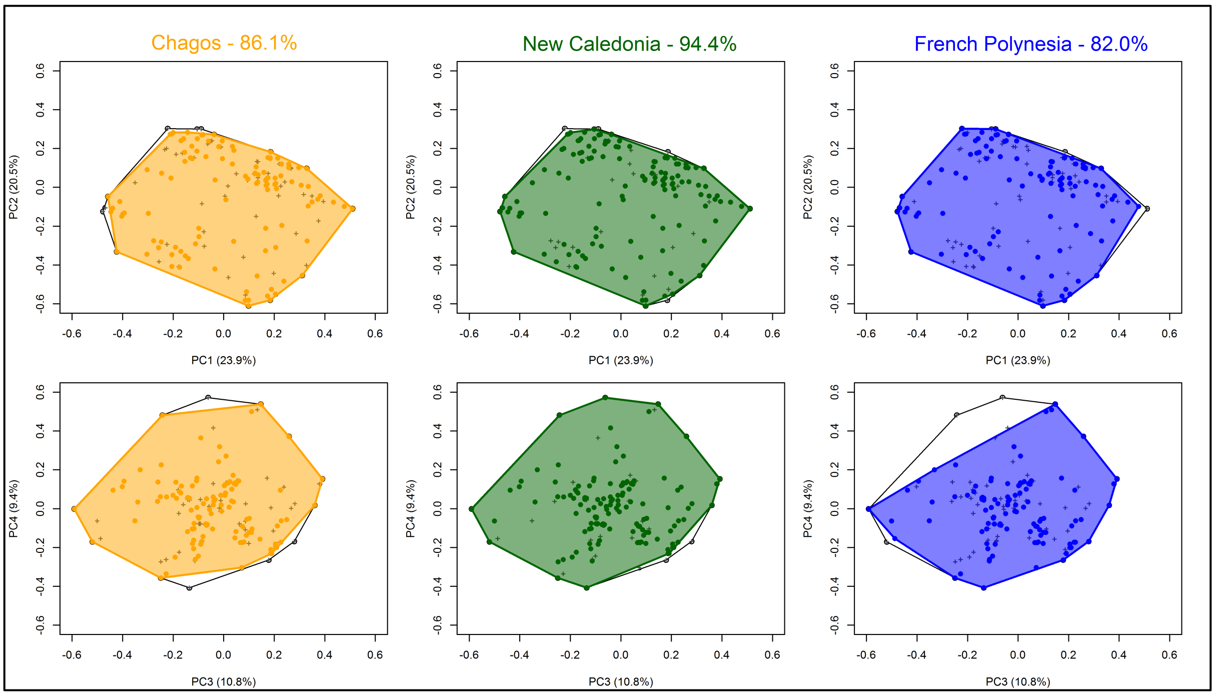


**Figure S4. Functional diversity of fish faunas in the three regions.**
The distribution of functional entities is represented in the same functional space where axes are PC1-PC2 and PC3-PC4 axes respectively, from a Principal Coordinate Analysis performed on a Gower distance between species pairs based on functional traits (*Material and Methods*). The global convex hull, corresponding to the total pool of 412 species regrouped into 157 functional entities is outlined in dark gray. The colored areas show the functional volume filled by each fish fauna in Chagos, New Caledonia and French Polynesia. Colored circles represent functional entities occurring in each region. Grey crosses are functional entities absent in the regions. The percentage of the global functional volume filled by the fish fauna of each region is given just after the name of the region.

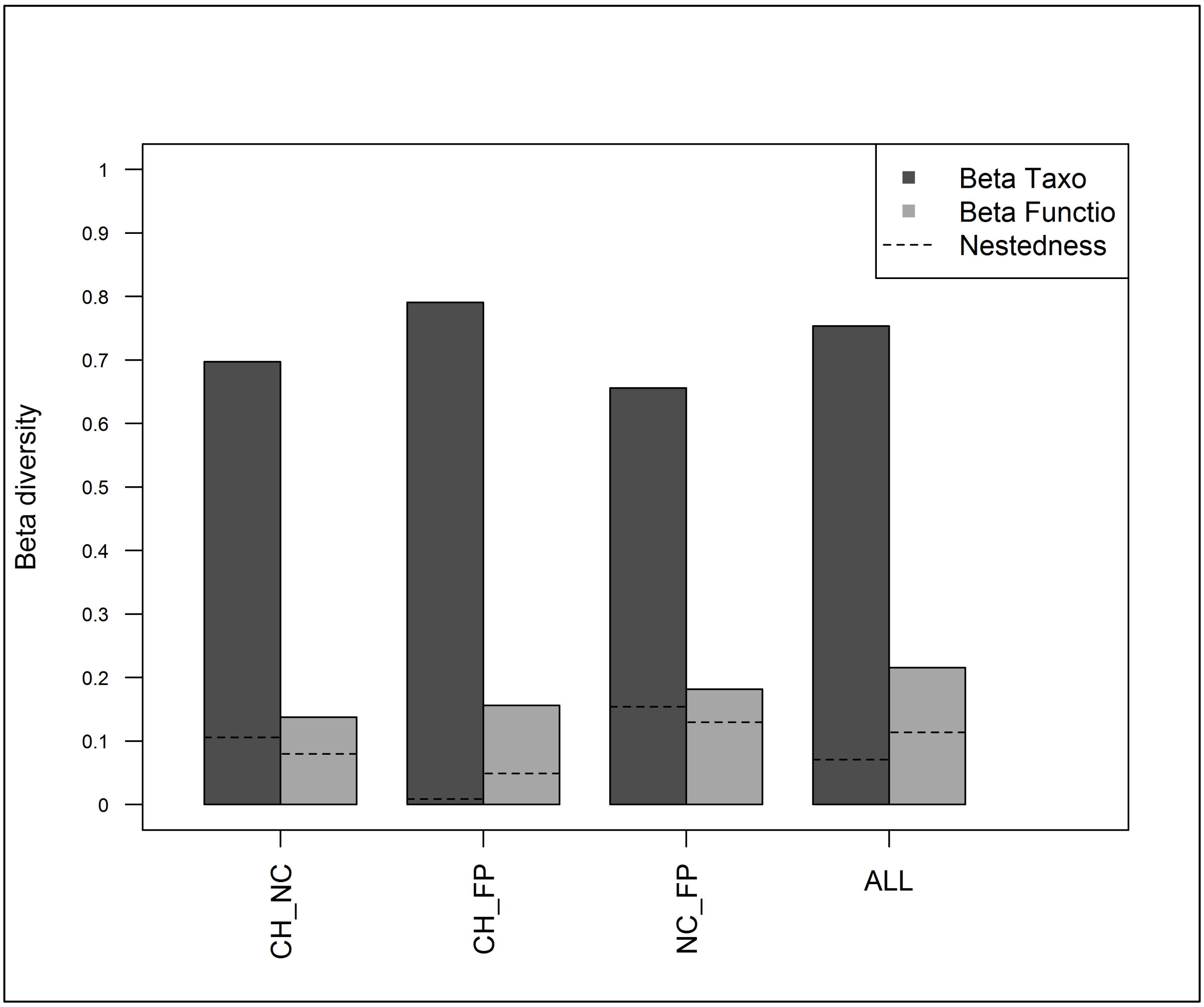


**Figure S5**. **Pairwise taxonomic and functional beta diversity**.
The taxonomic and functional beta diversity were computed using the partitioning framework of Baselga (2010) and Villéger et al. (2013) respectively, to measure the nestedness and replacement components of beta diversity. The replacement component implies that species are replaced by others between regions whereas the nestedness component relates the loss or gain of species between regions.
A high nestedness implies than the smallest species pool is a subsample of the largest whereas a high replacement component is due to 'true' species turnover between regions. See Table S1 for values. CH is for Chagos, NC for New Caledonia, FP for French Polynesia and TG for Tonga.

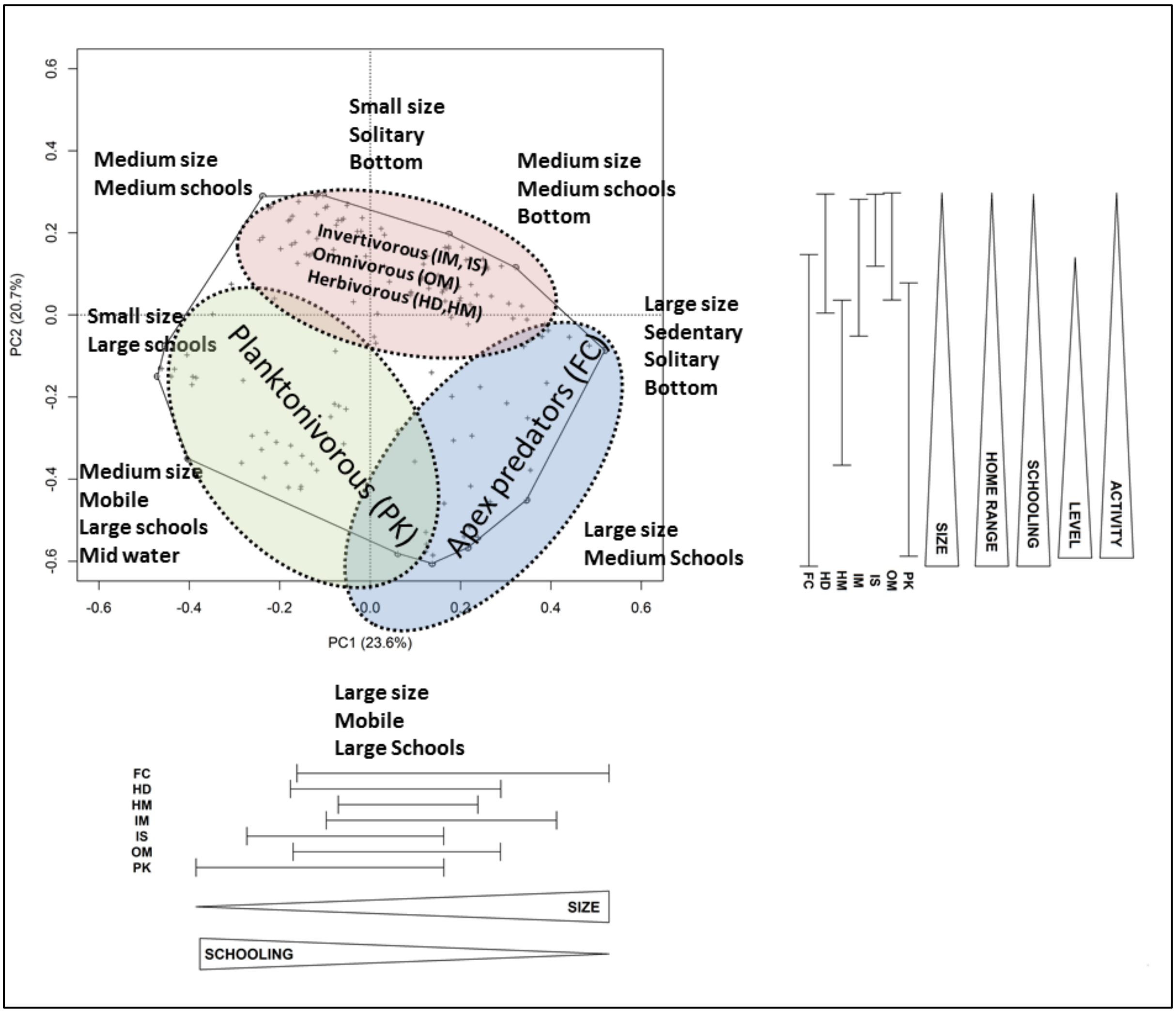


**Figure S6. Biological characterization of PCoAs axes**

Functional traits characterizing the two first dimensions of the functional space (PCoA axes 1 and 2): 1) "Size" of fish, 2) "Home-range" or the mobility of fish, 3) "Schooling" or the gregariousness of fish species, 4) "Level" or the vertical position in the water column and 5) the "Diet" with 7 categories (HD: herbivorous-detritivorous, HM:macroalgal herbivorous, IS: invertivorous targeting sessile invertebrates, IM: invertivorous targeting mobile invertebrate, PK: planktivorous, FC: piscivorous, and OM: omnivorous and 6) the "Activity" (diurnal or nocturnal).

The range coordinates of PCoA axes for the different modalities of ordered functional traits were used to define the trait modalities characterizing PCoA axis. For the "Size", "Home range", "Schooling", "Level" and "Activity" traits, the larger part of arrows indicate the highest values of the modalities. For the "Diet" trait, the range of the values is indicated for each modality.

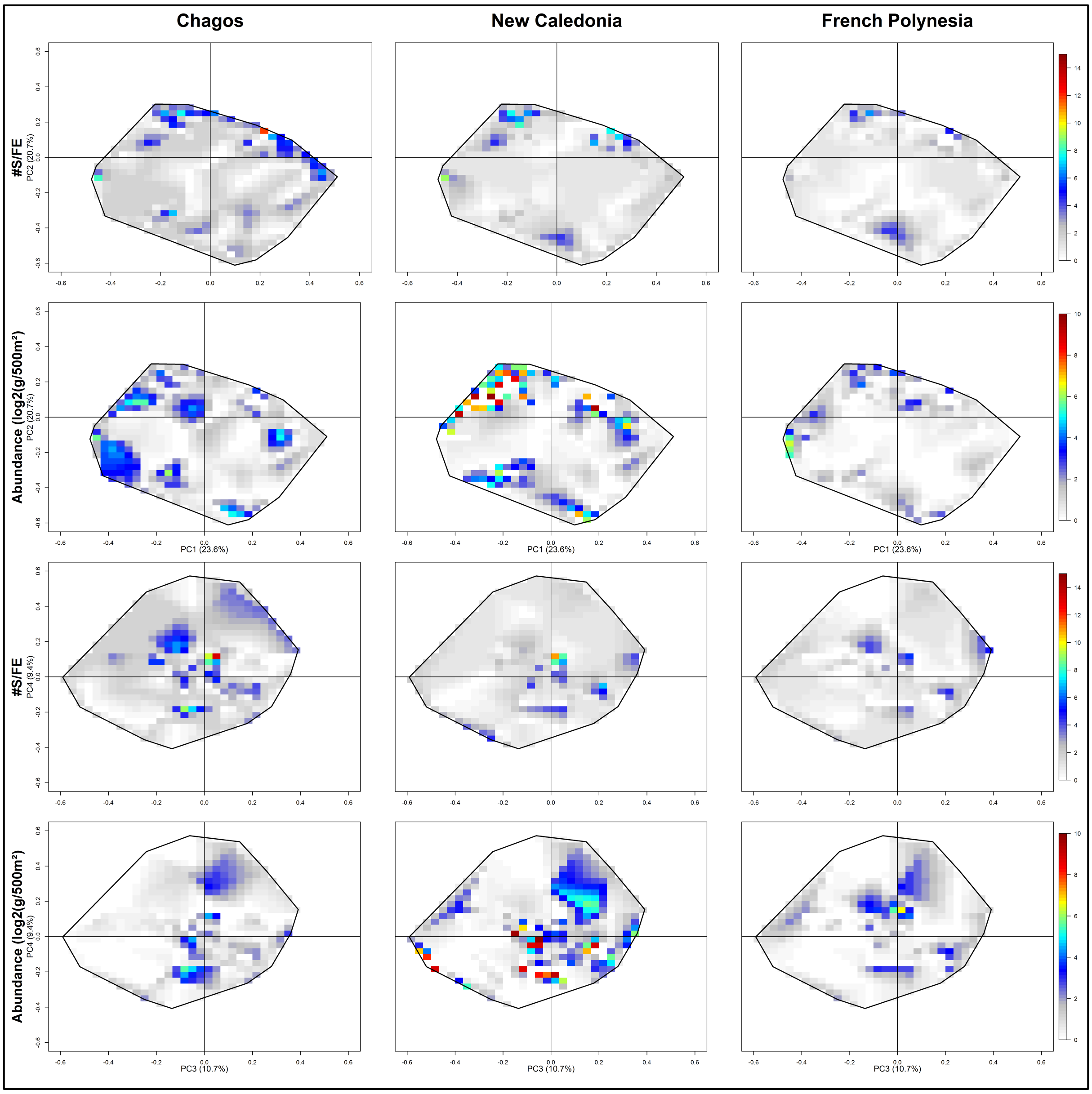


**Figure S7**. **Mapping of functional redundancy in the functional space.**

(a) shows the distribution of the number of species per functional entities in the PC1-PC2 functional space and (c) in the PC3-PC4 functional space.

(b) shows the distribution of the number of individuals per functional entities in the PC1-PC2 functional space and (d) in the PC3-PC4 functional space.

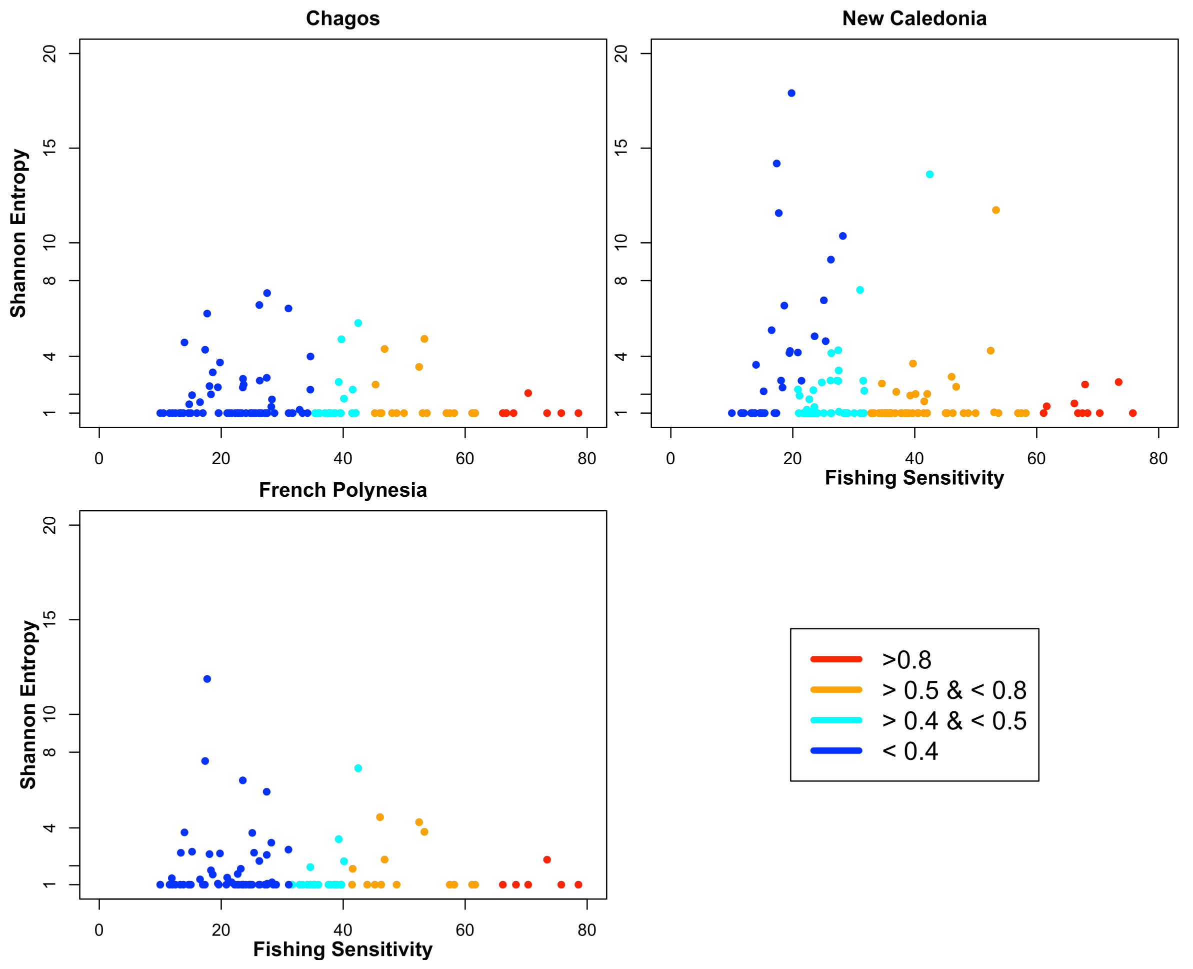


**Figure S8. Relationships between functional vulnerability and functional redundancy (Shannon entropy) for each region.**

Functional vulnerability is classified in four categories, from the highest (> 0.8, red) to the lowest (< 0.4, blue) in each plot a) Chagos, b) New Caledonia, c) French Polynesia.

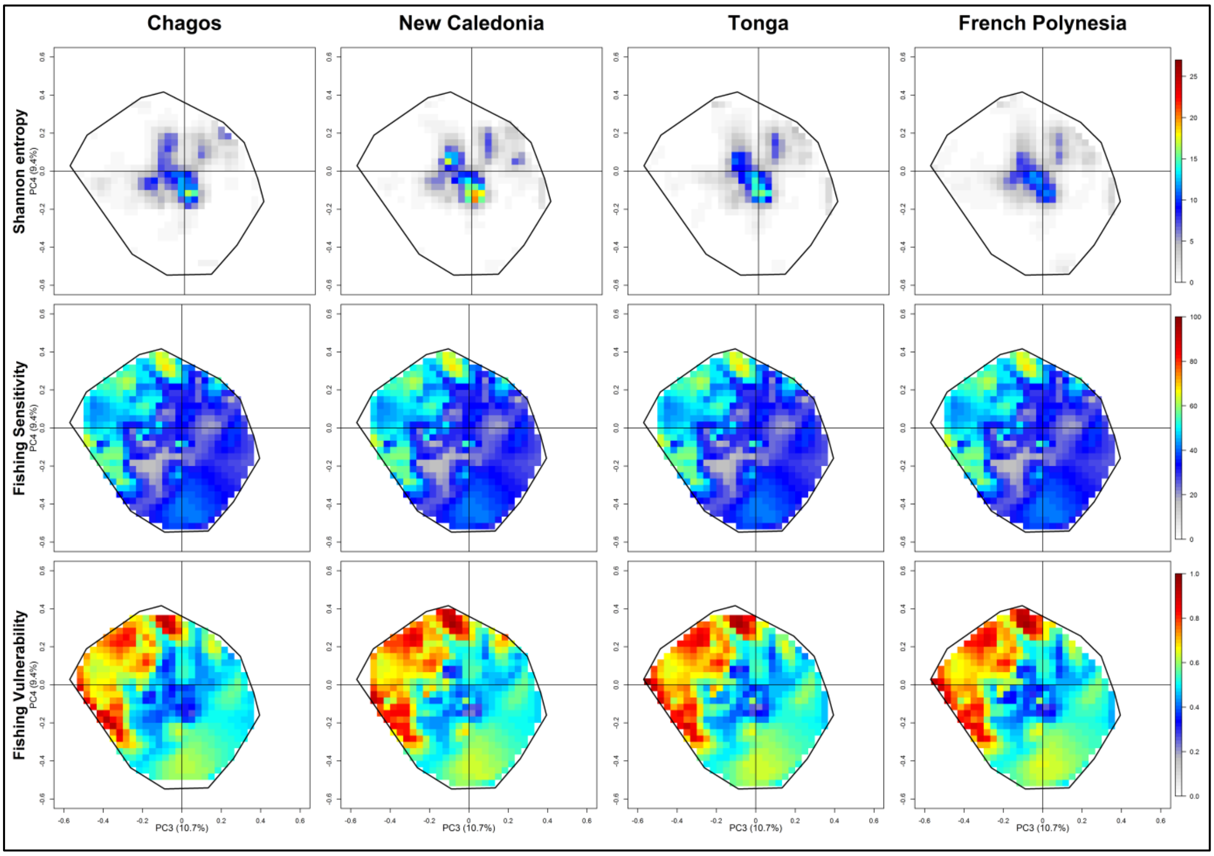


**Figure S9**. **Mapping of redundancy, sensitivity and vulnerability in the functional space in the PC3-PC4 functional place.**
The top row (a) shows the distribution of the Shannon entropy, measuring the level of functional redundancy in functional entities, across the functional space for each region.
The middle row (b) shows the distribution of sensitivity to fishing across the functional space while the bottom row (c) is the distribution of vulnerability to fishing in this space.

**Table S1**. Taxonomic (Dark Grey) and Functional (Light Grey) Beta diversities between pairs of regions.

| | Chagos | New Caledonia | French Polynesia |
|---|---|---|---|
| **Chagos** | - | 0.14 | 0.16 |
| **New Caledonia** | 0.70 | - | 0.18 |
| **French Polynesia** | 0.79 | 0.65 | - |
